# Predictive and mechanistic multivariate linear regression models for reaction development

**DOI:** 10.1039/c7sc04679k

**Published:** 2018-01-23

**Authors:** Celine B. Santiago, Jing-Yao Guo, Matthew S. Sigman

**Affiliations:** a Department of Chemistry , University of Utah , 315 South 1400 East , Salt Lake City , Utah 84112 , USA . Email: sigman@chem.utah.edu

## Abstract

Multivariate Linear Regression (MLR) models utilizing computationally-derived and empirically-derived physical organic molecular descriptors are described in this review. Several reports demonstrating the effectiveness of this methodological approach towards reaction optimization and mechanistic interrogation are discussed. A detailed protocol to access quantitative and predictive MLR models is provided as a guide for model development and parameter analysis.

## Abstract 2

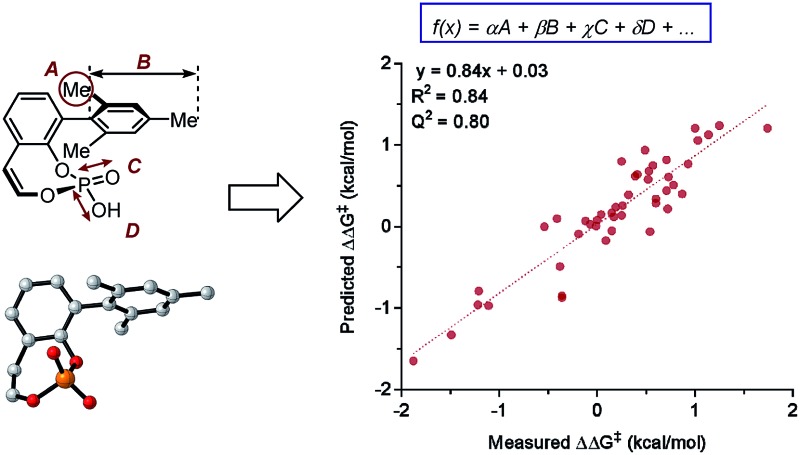
The utilization of physical organic molecular descriptors for the quantitative description of reaction outcomes in multivariate linear regression models is demonstrated as an effective tool for *a priori* prediction and mechanistic interrogation.

## Introduction

The development of a new reaction methodology, especially in asymmetric catalysis, can be a challenging and expensive task, as it is generally attained through exhaustive reaction screening.^1^ Traditional reaction optimization routes are often based on empiricisms with occasional systematic approaches such as Design of Experiments (DoE)^2^ or High Throughput Screening (HTS).^1^,^3^–^6^ Additionally, mechanistic analyses are typically performed subsequent to completion of reaction optimization where computational studies are supplemented for further refinement of chemical understanding.^7^

A reaction optimization strategy which simultaneously interrogates reaction mechanism and identifies better performers during the early stages of reaction optimization is an evolving approach towards meticulous design of new catalysts.^8^–^18^ In particular, an optimization method by Sigman and coworkers^8^ utilizes multivariate linear regression (MLR) models that are acquired based on a mathematical relationship of the experimental reaction outcome (*e.g.*, selectivity (enantio-, regio-, and chemo-), turnover number and turnover frequency (TOF),^19^,^20^ reaction rate,^21^ and yield^22^) as a function of both experimentally-derived and calculated physical organic molecular descriptors. Substandard results with low yield/low enantioselectivity, commonly omitted without further consideration in the conventional empiricism-driven optimization route,^23^ are utilized in this MLR approach to generate a diverse and wide-ranging data set for statistical analysis.^24^

In order to attain statistical models, it is a prerequisite to have structural modularity of the molecules of interest and consequently, large parameter libraries will need to be built. Recent advances in computational methods and resources encouraged the application of accurate molecular simulation utilizing density functional theory (DFT) to generate descriptors for molecular-feature-based MLR model applications. A notable advantage of this MLR approach over Quantitative Structure Activity Relationship (QSAR)^25^–^27^ is the selection and employment of physically meaningful molecular descriptors instead of topological descriptors.^28^ Therefore, useful mechanistic information can be gathered from well-validated mathematical models. In comparison with transition state analysis, the MLR approach has a substantially lower computational requirement since it utilizes ground state structures as the parameter source and an initial mechanistic hypothesis is unnecessary. Moreover, this computational advantage of the MLR approach provides means for virtual screening, where reaction outcomes can be predicted *a priori*.^29^,^30^ Application of these modern statistical analysis tools in asymmetric catalysis can accelerate reaction optimization and provide a platform for *de novo* catalyst design through predictive modelling.

The aim of this mini**review** is to demonstrate the capabilities of a predictive and mechanistically informative MLR modelling approach *via* utilization of suitable physical organic molecular descriptors. Additionally, a detailed protocol is provided describing the step-by-step process from parameter acquisition and selection, to multivariate linear regression.

## Molecular descriptors

Since the seminal work of Hammett in the 1930s, Linear Free Energy Relationships (LFERs) have been widely used by the organic chemistry community to relate structure to function with the purpose of gaining mechanistic information and predicting reaction outcomes.^31^–^35^ Recognizing the inherent ambiguity in qualitative evaluation of reactivity patterns based only on chemical structure, Hammett developed a quantitative molecular descriptor, *σ*, to describe aryl substituent electronic effects. The broad applicability of the Hammett parameter and the LFER method triggered the development of various molecular descriptors.^26^,^36^,^37^ In this section, physically meaningful molecular descriptors that have been applied in multivariate linear regression analysis are discussed.

### Steric parameters

Steric effects play a key role in asymmetric induction since the spatial orientation of every reactive species during the stereodetermining step must be precisely controlled. This prompted the generation of parameters to quantitatively describe steric effects. Various steric parameters that have been previously introduced in the literature include the Taft parameter,^36^ Charton parameter,^38^ Sterimol values,^39^ Tolman cone angle,^40^ buried volumes,^41^ torsion angles, bond lengths, and bite angles.^42^,^43^

#### Taft, Charton, and Sterimol values

**Fig. 1:**
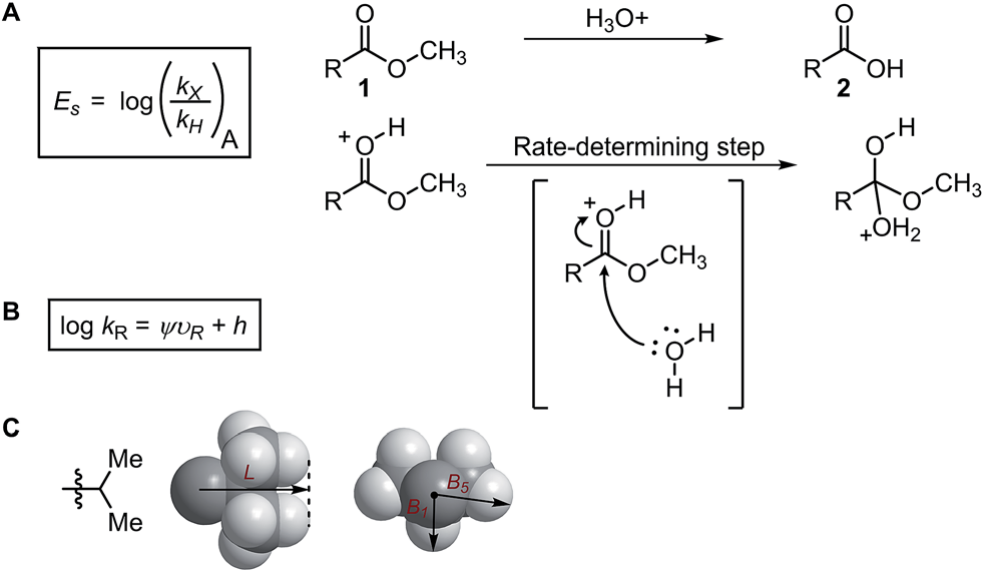
(A) Taft, (B) Charton, and (C) Sterimol steric parameters.

In the 1950s, Taft^36^ demonstrated that steric effects can be separated from electronic effects in the acid-catalysed hydrolysis of alkyl esters **1** delivering one of the first recognized steric parameters. The Taft steric parameter (*E*_s_) is calculated from the logarithmic value of the reaction rate of the substituted *versus* the unsubstituted methyl ester (Fig. 1A). The substituent-induced resonance and inductive effects are diminished since the charge formed during the rate-determining step is preserved.

A decade later after the introduction of *E*_s_, Charton proposed an improved variation of the Taft steric parameter, which further eliminates the electronic influence by correlating the experimentally measured reaction rates from the acid-catalysed hydrolysis with the calculated van der Waals radii (Fig. 1B).^38^ This experimentally verified parameter is called the Charton value (*υ*). Considering the multifaceted nature of steric effects, Verloop presented a more sophisticated set of steric parameters, the Sterimol values, which provides various dimensional measurements as subparameters instead of a single, cumulative value that represents the entire spatial information.^39^ The most representative Sterimol parameters include the distance along the bond axis *L*, the minimum radius perpendicular to the bond axis *B*_1_, and the maximum radius *B*_5_ (Fig. 1C).

**Fig. 2:**
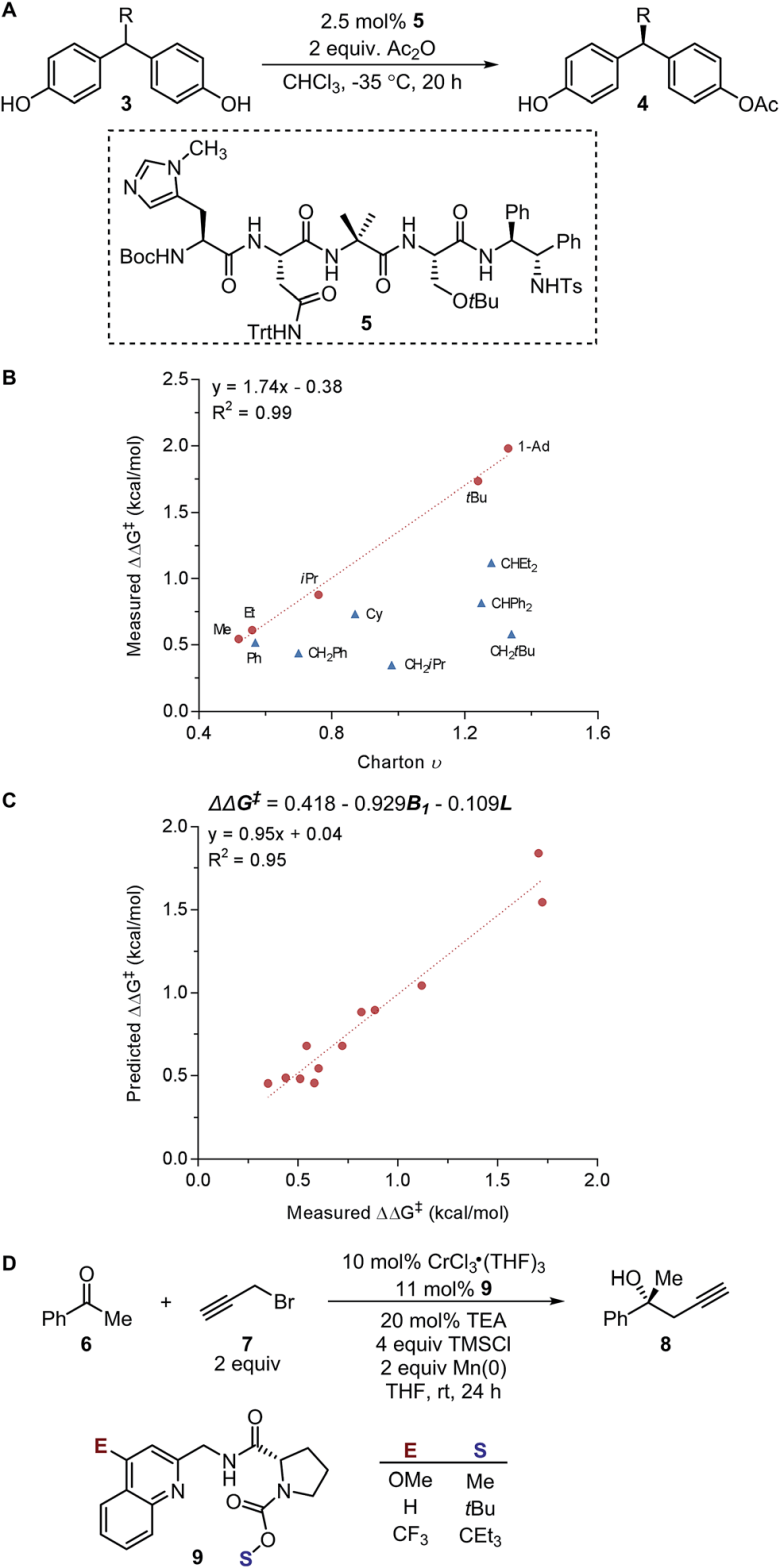
(A) Desymmetrization of bisphenol. (B) Charton–LFER model. (C) Sterimol–LFER model. (D) Nozaki–Hiyama–Kishi propargylation of acetophenone.

These physical organic steric parameters were initially developed for QSAR analysis in evaluation of biological activity, but were recently shown as valuable tools in asymmetric catalysis. A study by Harper *et al.* has compared the Charton and Sterimol steric parameters in an effort to quantitatively define the influence of the substituent steric effects on the enantioselectivity in the desymmetrization of bisphenol **3** using a peptide catalyst **5** as previously reported by Miller (Fig. 2A).^44^,^45^ The Charton value of the substituent was found to be inadequate in describing the steric influence from unsymmetrical substituents on the measured enantioselectivity (Fig. 2B). This break in linearity in the Charton LFER model exposed a potential deficiency of Charton values caused by its simplified treatment of substituents, which are considered as freely rotating groups and thus are described as spheres. In contrast, the dimensionality feature of the Sterimol values allows for a more detailed description of the substituent shape. Through multivariate analysis, a superior model was generated relating the observed enantioselectivity (ΔΔ*G*^‡^) to the *R* substituent Sterimol *B*_1_ and *L* values (Fig. 2C). A similar approach was presented in the analysis of enantioselective Nozaki–Hiyama–Kishi propargylation of methyl ketone **6**, where a multivariate linear regression analysis using a combination of Sterimol values derived from the quinoline-proline ligand **9** was able to depict the enantioselectivity (Fig. 2D).

**Fig. 3:**
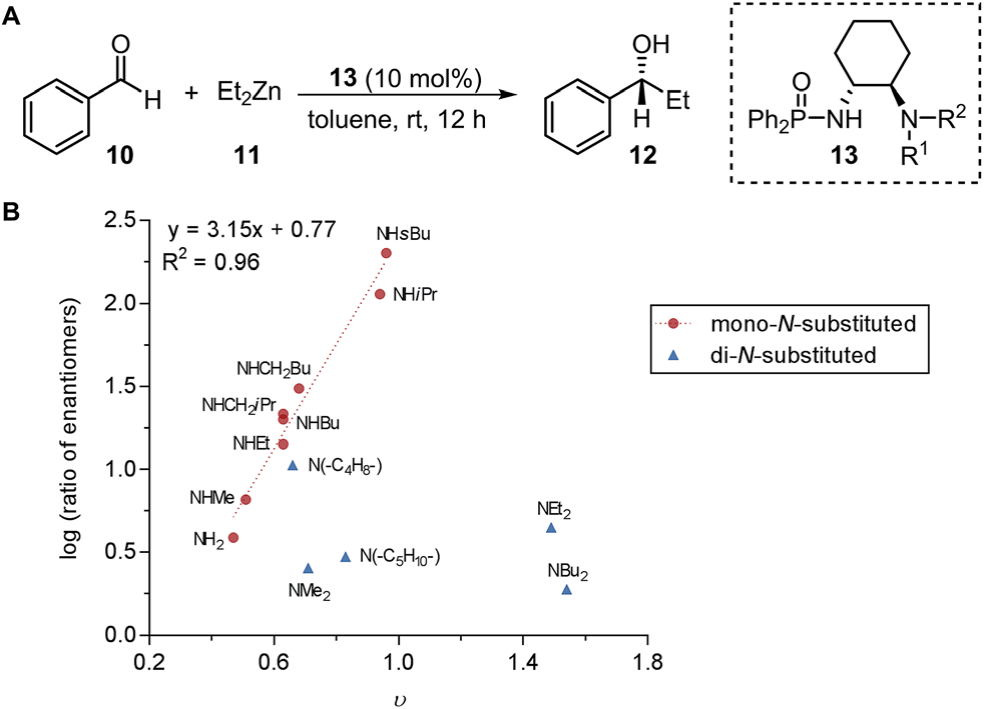
(A) Asymmetric addition of diethylzinc with benzaldehyde. (B) Charton-LFER model of mono- and di-*N*-substituted phosphoramide catalysts.

Subsequently, the Song laboratory investigated how the amino group of the chiral phosphoramide catalyst **13** affects the measured enantioselectivity in the asymmetric addition of diethylzinc **11** with benzaldehyde **10** (Fig. 3A).^46^ The Charton value *υ* of the amino substituent can account for only the enantioselectivity induced by mono-*N*-substituted catalysts, while the di-*N*-substituted chiral phosphoramide catalysts have to be excluded from the Charton-LFER model (Fig. 3B). This inability of the Charton parameter to describe the heterogeneity in the amino substituents further illustrates its limitations. In comparison, with the utilization of the individual Sterimol *B*_1_ values of the *R*^1^ and *R*^2^*N*-substituents as parameters, both the mono-*N*-substituted and di-*N*-substituted chiral phosphoramide catalysts were successfully incorporated in one comprehensive model. Additionally, Sterimol MLR models were utilized to depict the enantioselectivity invoked by chiral 1,2-amino-phosphinamide ligands in a Henry reaction^47^ and chiral 1,2-amino-phosphoramide ligands in the asymmetric addition of diethylzinc to acetophenone.^48^

#### Tolman cone angle and percent buried volume

**Fig. 4:**
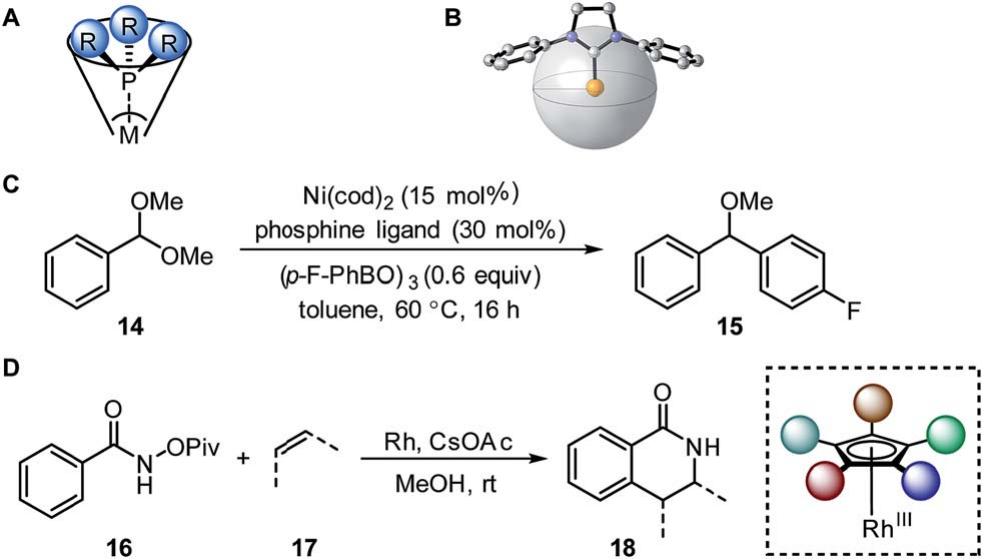
(A) Tolman cone angle. (B) Percent buried volume. (C) Nickel-catalysed Suzuki cross-coupling. (D) Rhodium-catalysed C–H activation.

Tolman introduced the cone angle as a steric metric for phosphine ligands based on space-filling models.^40^ The Tolman cone angle (*θ*) is the measured apex angle across the phosphorus atom by projecting an arbitrary cylindrical cone from the metal atom positioned at the vertex towards the edge atoms of the phosphinyl substituent positioned at the perimeter (Fig. 4A). The metal to phosphorus distance is usually set to a standard value of 2.28 Å, in agreement with the Ni–P bond length in [Ni(CO)_3_(L)] complexes. However, the Tolman cone angle is often unable to describe modern unsymmetrical and more structurally complex ligands, such as the Buchwald-type biarylphosphines, bidentate ligands, and *N*-heterocyclic carbenes.^49^ Inspired by the Tolman cone angle, Nolan and Cavallo reported the percent buried volume (%*V*_bur_) as a steric parameter to better represent the steric bulk of *N*-heterocyclic carbenes.^41^,^49^,^50^ The percent buried volume is defined as the percent of the volume that the ligand occupies in an abstract sphere with the metal atom positioned at the centre (Fig. 4B). Cavallo and coworkers developed a program called SambVca to calculate %*V*_bur_ from X-ray crystal structures and calculated geometry-optimized structures.^51^,^52^

A recent report by Wu and Doyle examined the influence of phosphine ligands on the yield of a nickel-catalysed Suzuki C-sp^3^ coupling of acetals **14** with boronic acids to generate benzylic ethers **15** (Fig. 4C).^22^ The Tolman cone angle and the percent buried volume of a variety of phosphine ligands (including Buchwald-type ligands) were compared in order to delineate the differences between the two steric parameters. After performing a MLR modelling approach, the remote steric hindrance as depicted by the high *θ* and low %*V*_bur_ of high-yielding phosphine ligands was identified as a critical factor to improve the reaction yield.

Since the Tolman cone angle is specifically designed to describe phosphines, extending the application of this steric readout to other ligand types would be relevant to further understanding of organometallic reactions. As a demonstration, Paton, Rovis, and coworkers employed cone angle and Sterimol analysis to rationalize how cyclopentadienyl (Cp^*x*^) ligands structurally affect the regioselectivity and diastereoselectivity in rhodium-catalysed C–H functionalization reactions (Fig. 4D).^53^

#### Torsion and bond angles

The angles are notable steric parameters owing to the simple fact that atoms proximal in space tend to repel each other. The bulkier *N*-heterocyclic carbene ligands results in wider angles due to the increased steric repulsion towards the alkylidene group in ruthenium-NHC complexes as reported by Jensen and coworkers.^54^ Picazo, Houk, and Garg identified that the alkyne terminus with the larger internal angle in DFT-optimized structures will have a higher propensity towards nucleophilic attack (Table 1).^55^ Consequently, the degree of distortion can be used to estimate the regioselectivity of the nucleophilic addition of arynes.

**Table 1:** Torsion angle analysis of benzynes

Aryne	Optimized structure	Angle difference	Regioselectivity
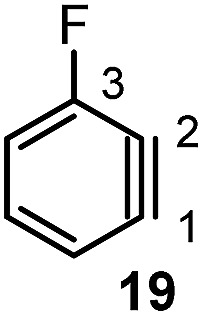	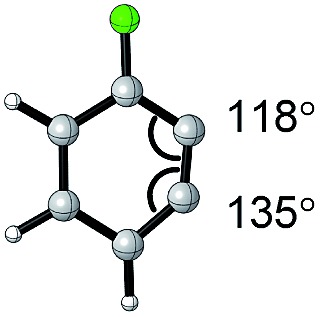	17°	C1 addition exclusive
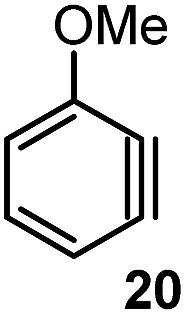	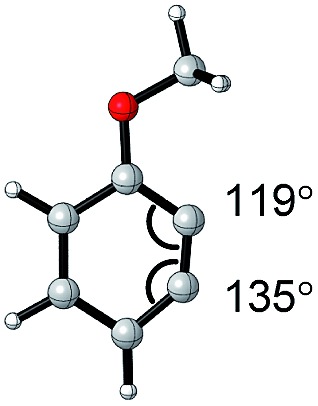	16°	C1 addition exclusive
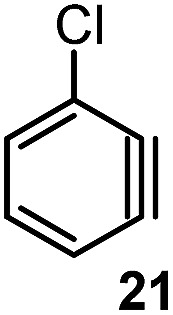	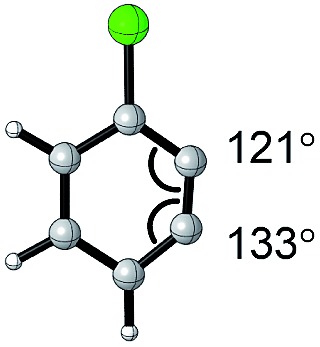	12°	C1 favored >20 : 1
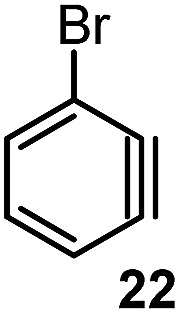	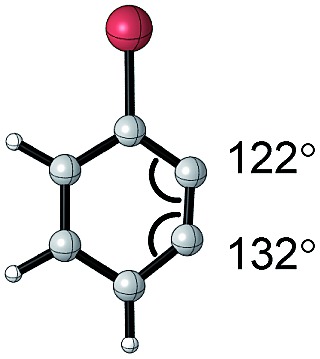	10°	C1 favored >13 : 1
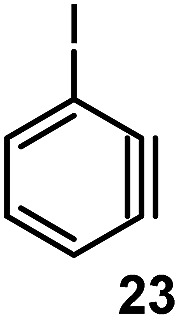	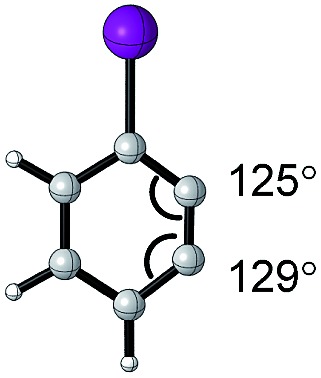	4°	C1 favored >9 : 1

### Electronic parameters

While asymmetric induction has been traditionally attributed to the steric influence of the chiral catalyst, remote variations altering the electronic properties of the catalyst can result in significant changes in enantioselectivity as well. With careful evaluation of ligand structure to activity, electronic manipulation of ligands can be an advantageous tool for design of asymmetric catalysts. In this section, various electronic parameters and their applications in LFERs will be discussed. It is noteworthy that, instead of representing purely electron density, most of these parameters incorporate structural information as well.

#### Hammett parameter

**Fig. 5:**
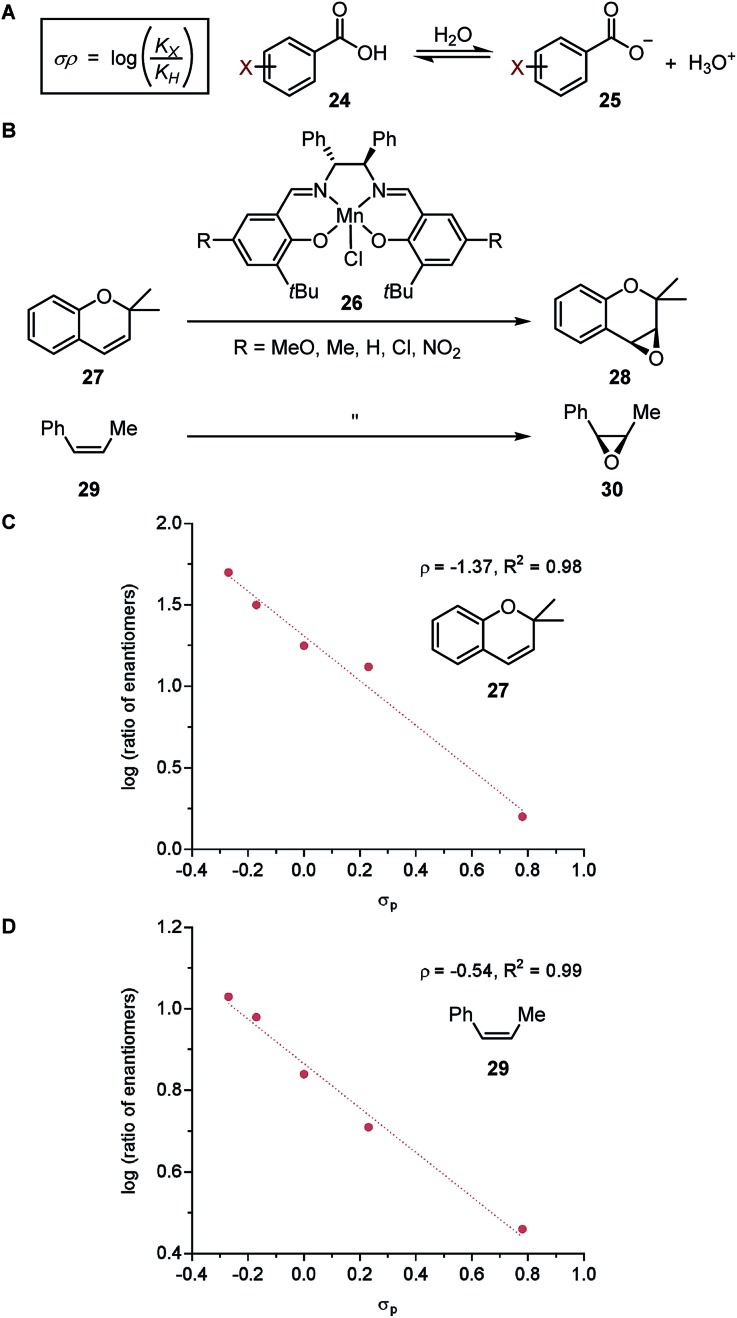
(A) Hammett parameter. (B) Enantioselective alkene epoxidation reactions. (C) LFER model for epoxidation of 2,2-dimethylchromene **27**. (D) LFER model for epoxidation of *cis*-β-methylstyrene **29**.

As discussed in an earlier section, the Hammett parameter (*σ*) is a quantitative measure of electronic effects for various *para*- and *meta*-substituents on the benzene ring.^31^–^33^,^35^ The work of Hammett is a pioneering example of LFERs, wherein the p*K*_a_ values of benzoic acid derivatives **24** were related to equilibrium constants (Fig. 5A) and reaction rates of various arene systems. The reaction constant, *ρ*, relates the log of equilibrium constants to the Hammett value, allowing comparison of substituent sensitivity to the standard set by the ionization of benzoic acid.

In a seminal report, Jacobsen and coworkers demonstrated that the manganese-salen catalyst **26** was highly sensitive to the remote electronic influence of the *para*-substituents in the enantioselective alkene epoxidation (Fig. 5B).^56^ Depicted by a correlation between the logarithmic values of the enantiomeric products and *σ*, a pronounced trend was revealed where manganese-salen catalyst **26** with electron-donating *para*-substituents resulted in higher enantioselectivities in the epoxidation reaction of 2,2-dimethylchromene **27** (Fig. 5C) and *cis*-β-methylstyrene **29** (Fig. 5D). The aryl substituent presumably affects the reactivity of the Mn-oxo intermediates, wherein an electron-donating group generates a milder oxidant resulting in a comparatively late transition state and thus, higher enantioselectivity.^57^

#### Infrared (IR) frequencies and intensities

Jones and coworkers demonstrated in 1957 that the IR carbonyl stretching frequency of acetophenone derivatives, with various substitutions at the *para* position of the phenyl ring, correlates well with the Hammett parameter.^58^,^59^ Furthermore, the classic Tolman electronic parameter (TEP) is determined from the *A*_1_-symmetrical CO stretching frequency of [Ni(CO)_3_(L)] complexes. It is used to quantitatively define the electron-donating or withdrawing ability of phosphine ligands.^40^

**Fig. 6:**
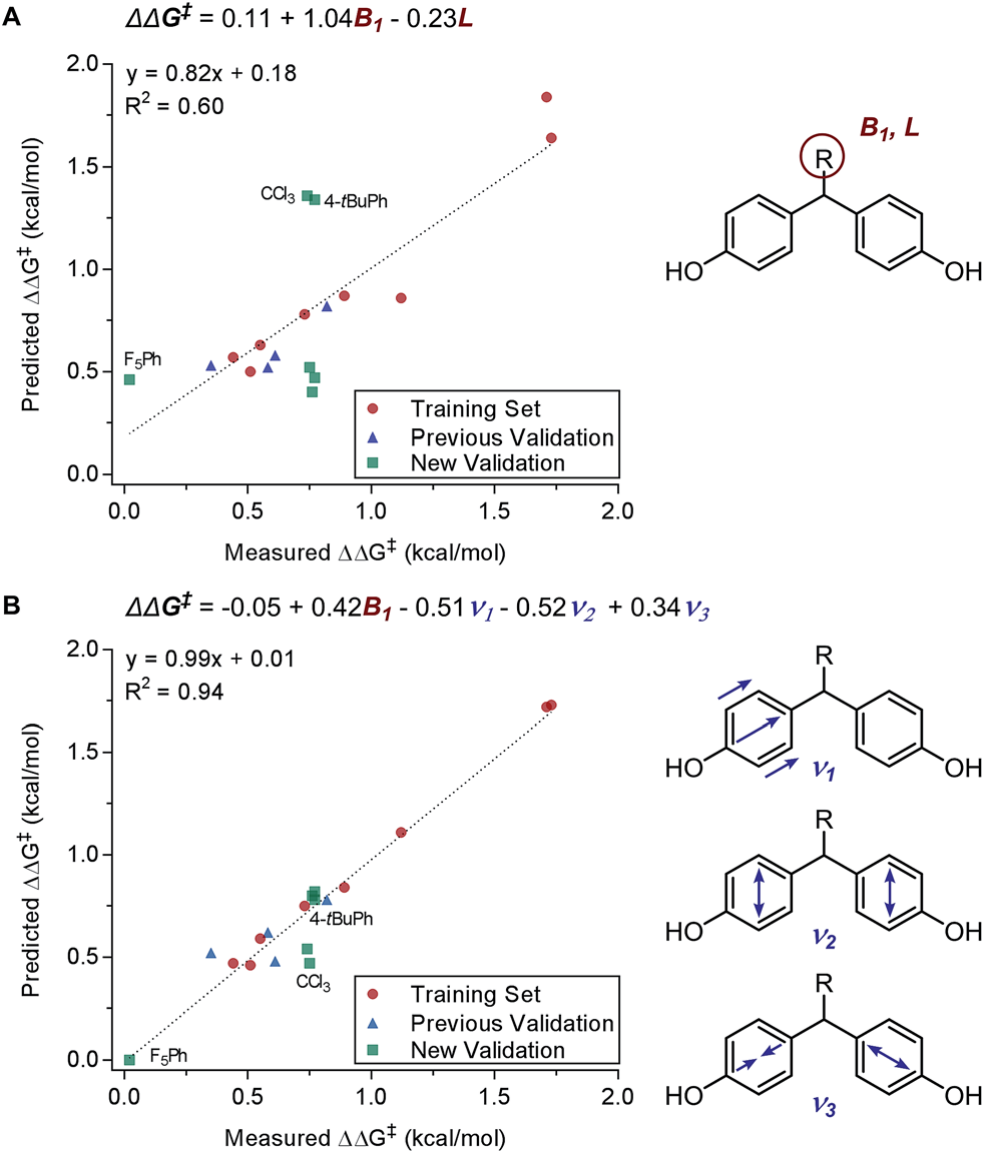
(A) Sterimol MLR model for desymmetrization of bisphenol. (B) IR stretching frequency MLR model for desymmetrization of bisphenol.

Principally, IR frequencies and intensities are considered to be stereoelectronic in nature as the molecular vibrational modes are directional changes dependent on mass and charge of the atoms in the molecule.^60^ Sigman and coworkers have extensively exploited the nature of IR frequencies and intensities in various case studies.^19^,^61^–^66^ As an example, the desymmetrization of bisphenol **3** was studied (Fig. 2A), wherein the Sterimol-MLR model failed to describe the enantioselectivity. Specifically, sterically bulky and electronically disparate bisphenol *R* substituents (CCl_3_, 4-*t*BuPh, and F_5_Ph) were shown to fail in the correlations (Fig. 6A).^61^ Through the employment of infrared-derived parameters from the bisphenol ring vibrations, steric and electronic effects were simultaneously depicted leading to improved validations (Fig. 6B).

#### Atomic charges

**Fig. 7:**
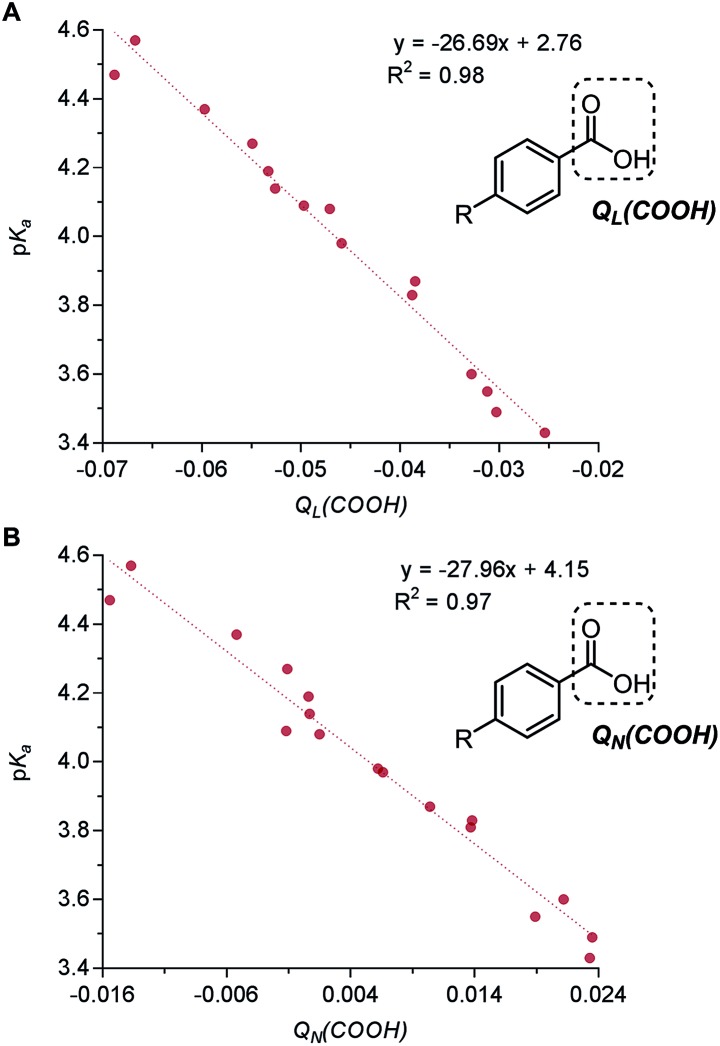
(A) Correlation of benzoic acid p*K*_a_ with benzoic acid group Löwdin partial charge *Q*_*L*_*(COOH)*. (B) Correlation of benzoic acid p*K*_a_ with benzoic acid group natural population analysis (NPA) partial charge *Q*_*N*_*(COOH)*.

Assigning charges to atoms has been a significant tool to understand reactivity in chemical reactions as well as electronic properties pertaining to dipole moments and nuclear magnetic resonance (NMR) chemical shifts.^67^ Since the designation of atomic charges involves the arbitrary partitioning of electron density distribution among the atoms in a molecule, it is hardly a proper quantum chemical property, and empirical validation is imperative to support this simulated feature. In a compelling investigation by Seybold and coworkers, the Löwdin (*Q*_*L*_*(COOH)*, Fig. 7A) and natural population analysis (NPA) atomic charges (*Q*_*N*_*(COOH)*, Fig. 7B) calculated from both the carboxylic acid group of various benzoic acids correlated well with the p*K*_a_ values.^67^,^68^ This relationship was afterwards extended further to a larger-sized panel of benzoic acids by Santiago *et al.*^63^ Additionally, White and coworkers demonstrated that MLR models of NPA charges and Winstein-Holness A-values^69^ were able to help predict the regioselectivity in C–H oxidation of (–)-triacetoxy calisiolide B.^70^

**Fig. 8:**
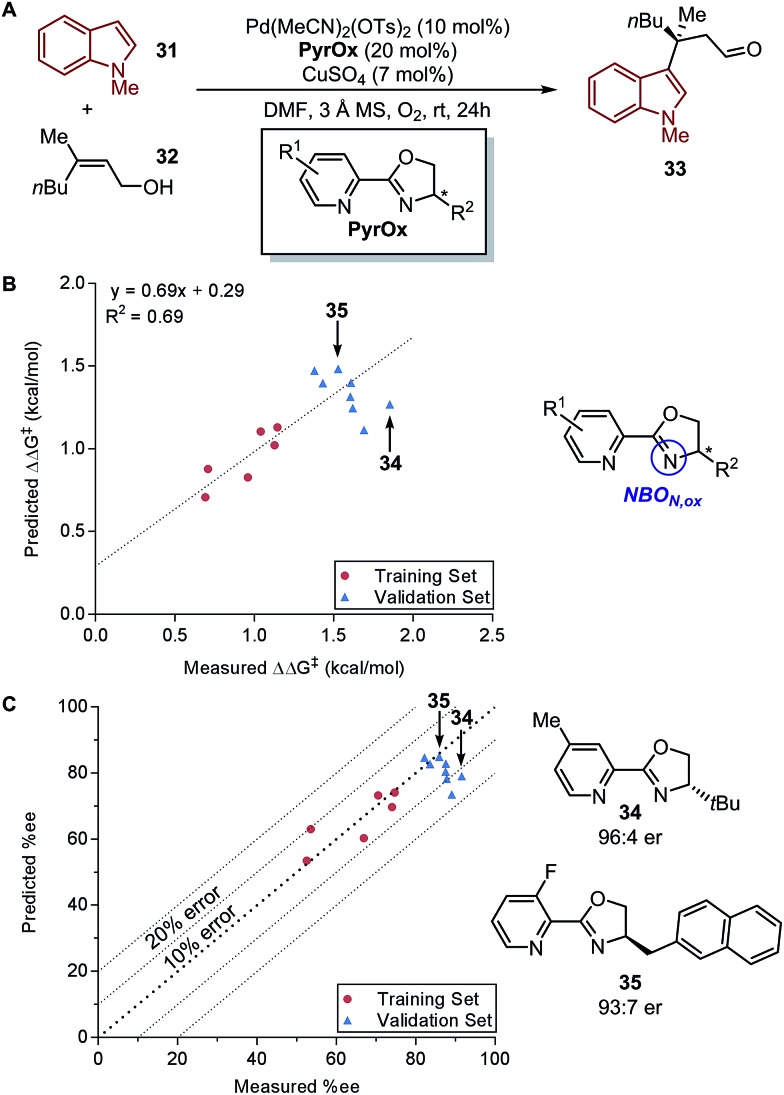
(A) Dehydrogenative Heck arylation of indoles with *cis*-alkenols. (B) Predictive model of enantioselectivity based on *NBO*_*N,ox*_. (C) Predictive model represented in %ee.

In a recent report by Zhang *et al.*, the natural bond orbital charge of the oxazoline nitrogen (*NBO*_*N,ox*_) in the pyridine-oxazoline (PyrOx) ligand was found to have a significant correlation with the enantioselectivity in a palladium-catalysed dehydrogenative Heck arylation reaction between indoles **31** and *cis*-alkenols **32** (Fig. 8A).^71^ Remote electronic effect was surveyed through varying the substitutions on the pyridine ring that modulates the *NBO*_*N,ox*_. Virtual screening was carried out based on this finding to reveal a set of superior ligands (Fig. 8B), which were within reasonable %error in terms of %ee (Fig. 8C).

#### NMR chemical shifts, coupling constants, and shielding tensors

NMR spectroscopy is one of the most reliable characterization tools to determine molecular structure.^72^ NMR-based parameters such as chemical shifts (*δ*), coupling constants (*J*), and shielding tensors (*σ*_xx_, *σ*_yy_, *σ*_zz_) can be obtained experimentally or computationally as potential molecular descriptors. As such, *δ* values relies on the molecule's orientation with respect to the external magnetic field, and varies depending on the steric and electronic environment surrounding the nucleus imparting knowledge of molecular functionality.^73^ In a report by Baran and coworkers, ^13^C NMR *δ* values were used to evaluate the preference for electrophilic oxidation of the tertiary C–H bonds and thus, predict the regiochemical outcome of the reaction.^74^ As there is an abundance of C–H bonds, predicting the regioselectivity in late stage C–H functionalization processes based only on chemical intuition is a difficult task, which highlights the benefit of quantitative prediction using NMR-derived parameters. In addition, NMR spin–spin coupling constants (*J*) embody information regarding bond distances, bond angles, and molecular connectivity.

**Fig. 9:**
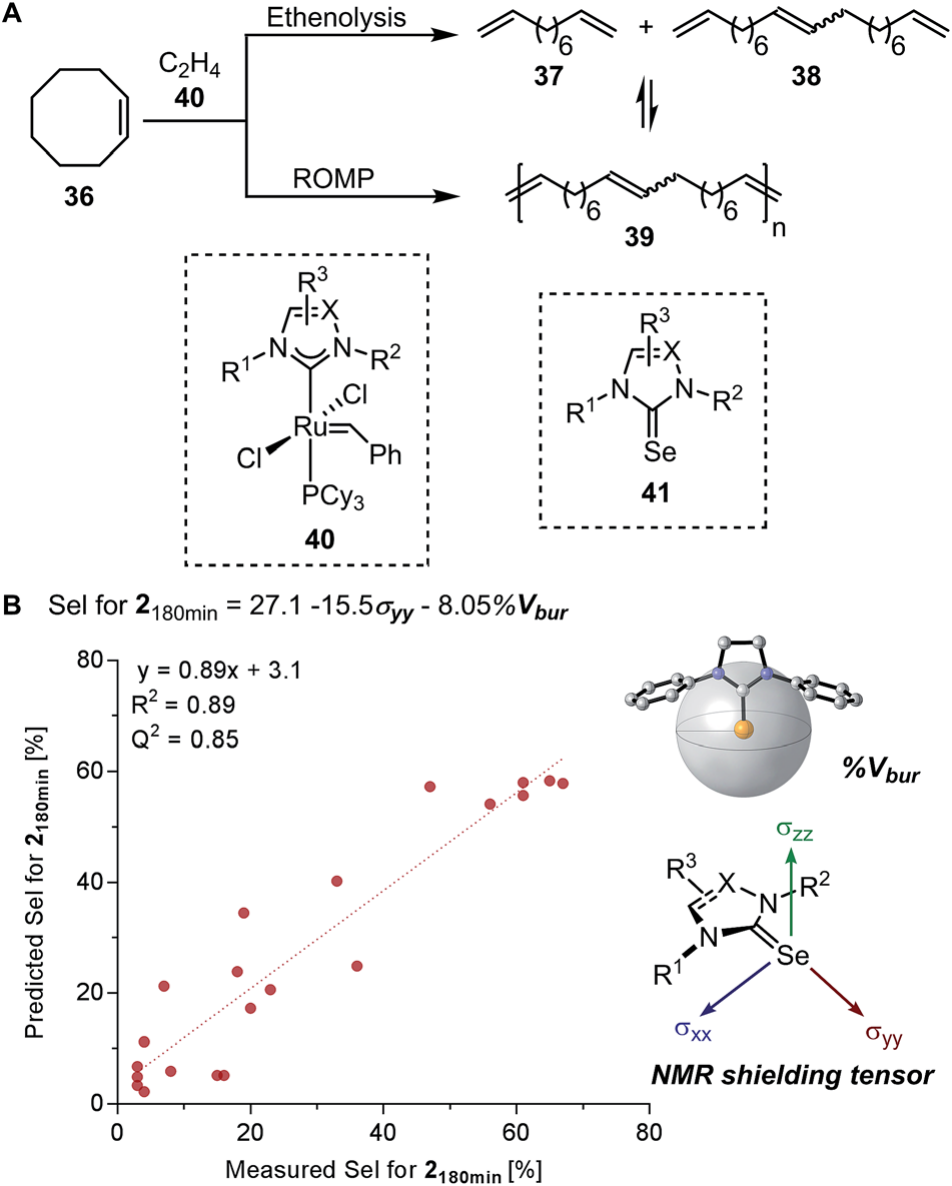
(A) Ruthenium-catalysed olefin ethenolysis and ring opening metathesis polymerization (ROMP) of *cis*-cyclooctene. (B) Ethenolysis selectivity model of NMR principal component tensor *σ*_yy_ and percent buried volume %*V*_bur_.

Based on the chemical shift anisotropy (CSA), the isotropic chemical shift (*δ*_iso_) is a rank-2 tensor which is defined as the average of the principal components of the chemical shift tensor (*δ*_xx_, *δ*_yy_, and *δ*_zz_).^75^,^76^ The directional information made accessible by the shielding tensor makes it a potentially more sophisticated molecular descriptor than the isotropic chemical shift. In 2008, Autschbach applied two-component (spin–orbit) relativistic density functional theory analysis method established on relativistic natural localized molecular orbitals (NLMOs) and natural bond orbitals (NBOs) to *δ* and shielding tensors.^77^–^79^ The extended application of this method, referred to as natural chemical shift (NCS) analysis, can indicate specific orbitals that have the highest impact on *δ*.^80^–^82^ Raynaud, Copéret, Eisenstein, and coworkers effectively utilized the NCS method *via* an orbital analysis of chemical shift tensors to identify precise fingerprints that distinguish between Fischer and Schrock carbenes.^83^ In a collaborative effort by Copéret, Sigman, and Togni groups on the study of ethenolysis of *cis*-cyclooctene **36** catalysed by a library of homologous [Ru–NHC] complexes **40** (Fig. 9A), the shielding tensor *σ*_yy_ component of the computed ^77^Selenium NMR chemical shift in [Se–NHC] complexes **41**, adducts of [Ru–NHC] complexes, was found to be correlative with the selectivity for ethenolysis (Fig. 9B).^84^ Through NCS analysis, it was identified that the *σ*_yy_ chemical shielding tensor is a probe of the π-backbonding ability of the NHC ligand.

#### Redox potential

**Fig. 10:**
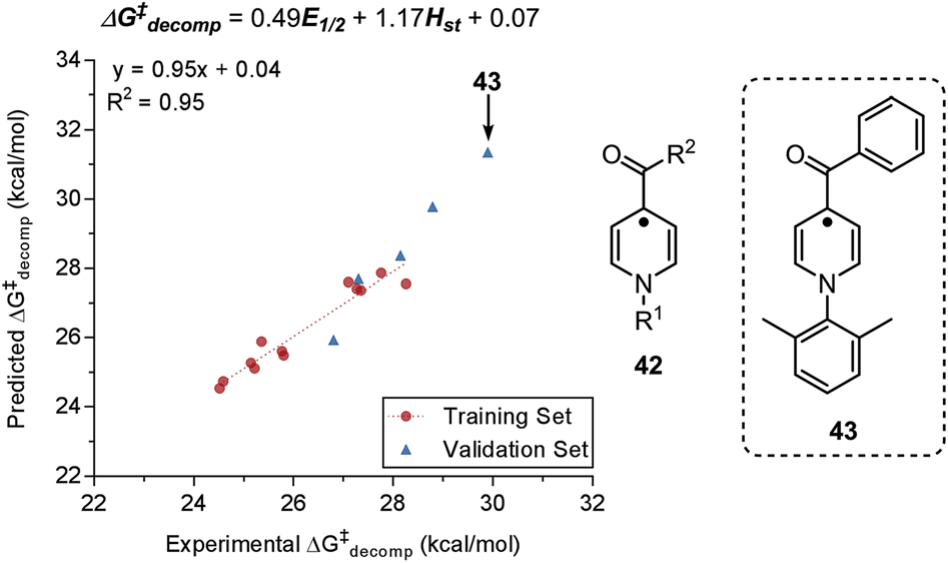
Predictive model for decomposition of pyridinium anolyte in relation to redox potential *E*_1/2_ and steric parameter *H*_st_.

The ability of a particular chemical species to gain or lose electrons can have direct impact on reactivity. The half-wave potential (*E*_1/2_) is defined as the propensity of a chemical species to be reduced, and this electrochemical measurement can easily be obtained from voltammetry experiments.^53^,^62^ Minteer, Sigman, Sanford, and coworkers generated a predictive multivariate model to assess the stability of pyridinium anolytes **42** for redox flow battery storage applications (Fig. 10).^21^ The decomposition barrier (Δ*G*^‡^) was evaluated as a function of the half-wave potential (*E*_1/2_) and the steric parameter, substituent height out of the pyridine ring plane (*H*_st_), as predictor variables (Fig. 10B). The obtained MLR model guided the design of a highly persistent *N*-xylyl-substituted pyridinium **43** as organic anolyte material. The high persistence of the identified pyridinium presumably results from the protection of the pyridine C2 and C6 positions by the xylyl substituent, which decelerates the undesired homo-coupling of the two pyridine radicals.

#### Non-covalent interaction (NCI) parameters

**Fig. 11:**
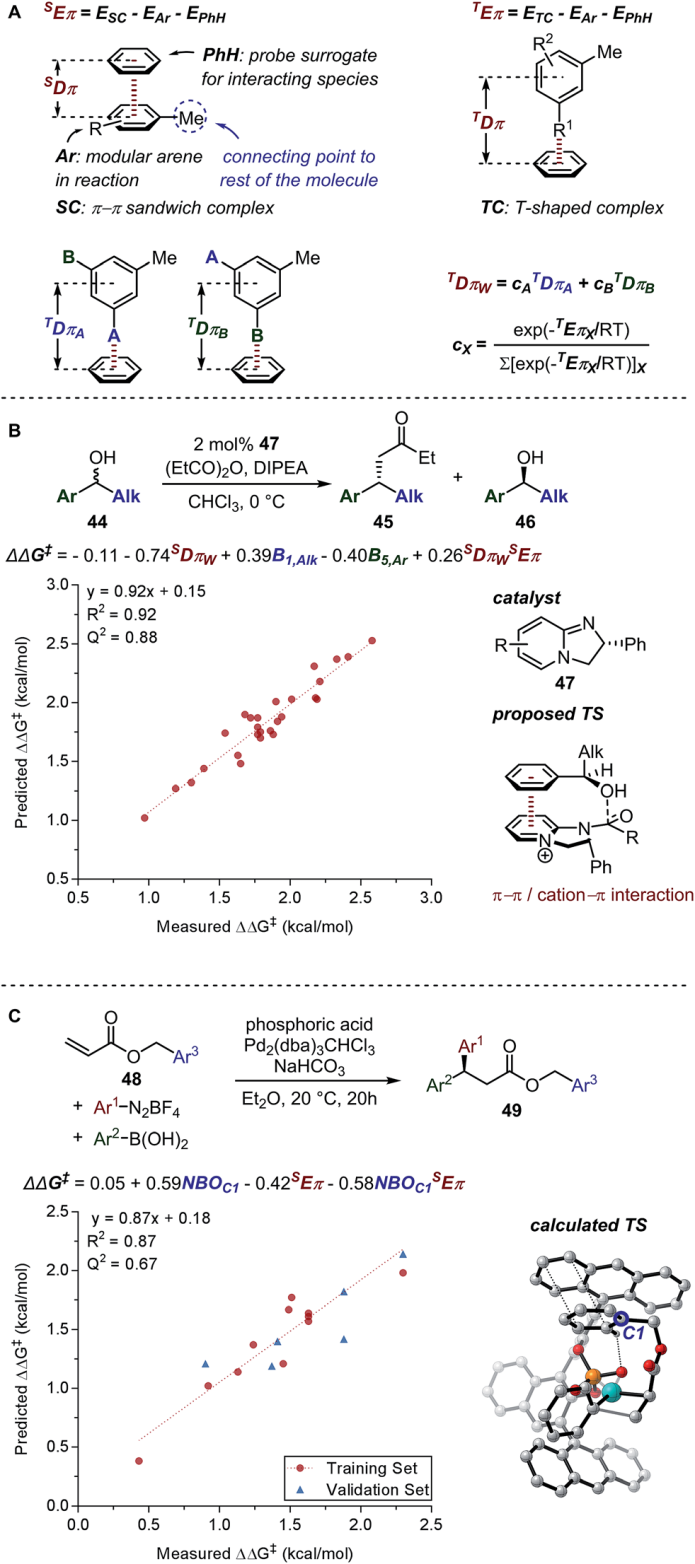
(A) *E*π_w_ and *D*π_w_ parameters. (B) Birman's kinetic resolution (C) palladium-catalysed 1,1-diarylation.

The interplay of distinct non-covalent interactions (NCI) between reaction participants orchestrates the selectivity attained in various catalytic processes.^85^–^87^ However, quantitative empirical descriptors for these NCIs are lacking due to the relatively small energy window (0–2 kcal mol^–1^) and the dynamic nature of this type of interaction.^86^ Thus, NCI parameters that are computationally-derived provide an attractive alternative. Taking an inspiration from the earlier work of Wheeler and Houk^88^ where relative π-stacking interaction energies (*E*_int_) between two interacting aromatic moieties were found to be correlative to Hammett *σ*_m_ parameter, new weighted NCI parameters were developed by Orlandi *et al.*,^89^ represented as *E*π_w_ and *D*π_w_ (Fig. 11A). These new parameters were defined as the Boltzmann averages of features from multiple potential conformers. Utilizing such descriptors in the multivariate linear regression analysis of Birman's kinetic resolution^89^ of benzylic alcohol **44** (Fig. 11B) and the palladium-catalysed 1,1-diarylation^90^ of benzyl acrylate **48** (Fig. 11C) suggested that the specific π-interactions are relevant in invoking enantioselectivity.

### Multivariate model development workflow

**Fig. 12:**
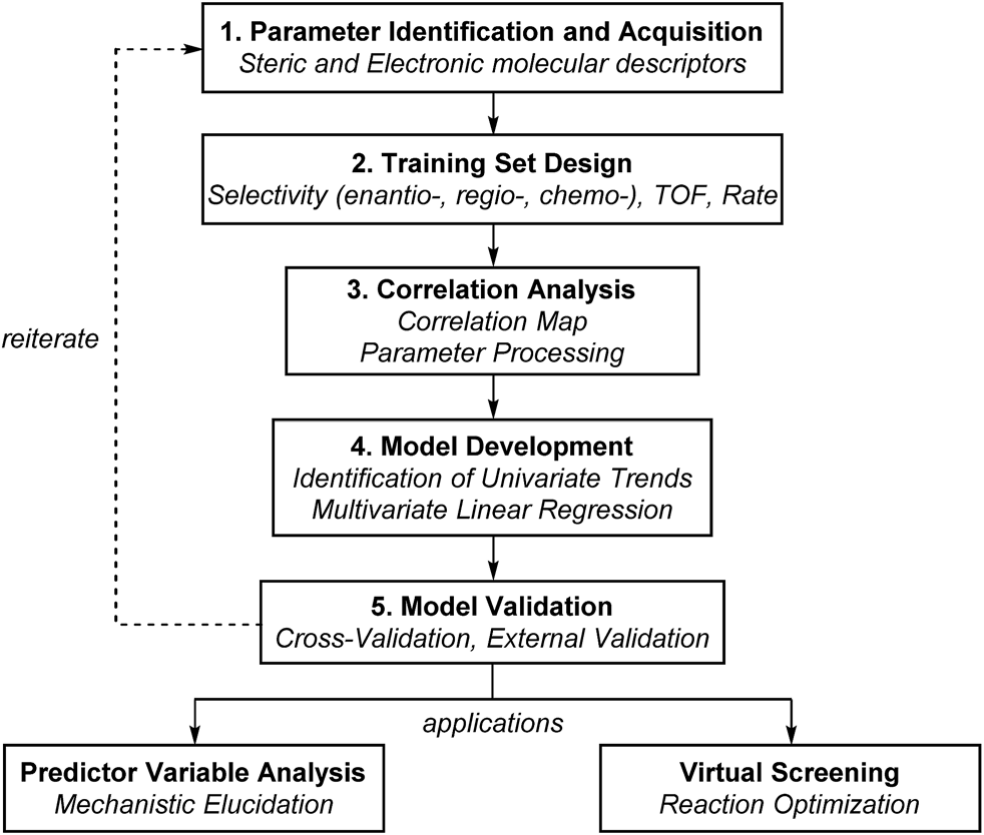
General scheme of model development.

The general protocol to generate multidimensional descriptive models is shown in Fig. 12. In this process, the major components involved are (1) the identification and acquisition of relevant parameters; (2) the design of an initial set of data for model construction (*i.e.*, the training set); (3) intercorrelation assessment; (4) preliminary model development involving identification of univariate trends and execution of multivariate linear regression; and (5) validation of multivariate models through cross- and external validation methods. Successful development of accurate, informative models should allow virtual screening to accelerate reaction optimization and predictor variable analysis to obtain mechanistic insights. In this section, a detailed guideline of each step for model construction and evaluation is provided.

### Parameter identification and acquisition

As discussed in the former section, a set of descriptive features needs to be selected and acquired, preferably from simulated structures with a well-balanced computational requirement and accuracy.^91^ Existing mechanistic knowledge of the reaction can guide parameter selection.

### Training set design

For the construction of generalizable, unbiased models,^92^–^94^ which are aimed at making accurate predictions for a range of molecules with considerable variations, instead of explaining only the data at hand, it is common to divide the acquired experimental data into two sets: a training set, which is used for model construction, and an external validation set, which is necessary for verification of the generated models.^95^–^97^ This arrangement allows for an efficient evaluation of model generalizability.

However, for the development of catalytic systems, in most cases, the number of observations may be quite limited (less than a hundred) by a statistical standard. Consequently, the modelling outcome can be highly dependent on the selected set for model training. Thus, the training set should be designed carefully to represent the entire poll of choices for the system under study. The selection of structurally diverse and well-distributed samples that encompass a wide range of reaction outcomes is a key element in training set design, which is crucial for the resulting models to be generalizable towards structural variations and relative accuracy in extrapolation.^98^ Countering the intuition of looking for the best possible results, the entries with low performance are equally important in this operation.^11^

Training set design requirements can be met in multiple ways. The first option is to base the selection on the knowledge of chemical structure, which though not quantitative, would be intuitive for a trained chemist, and is generally effective for modular structures.^99^ The second method is to perform a D-optimal design^100^ on a set of relevant parameters,^101^,^102^ which aims for maximum coverage of the sample space, as briefly demonstrated by Bess *et al.* in their analysis of the enantioselective NHK propargylation of alkyl ketones, where the training set was designed based on the evaluation of the presumed most important steric and electronic parameters.^103^ This method requires the front-end construction of a large virtual library, the corresponding comprehensive parameter set, and an initial guess of the relevant, influential parameters based on chemical knowledge and mechanistic speculation. This option is especially suited for model-guided screening where the collection of experimental results arise from the training set design, similar to the Design of Experiments (DoE) process.^104^ The third option, in contrast, is suited when modelling is performed at a late stage of screening, which involves selecting the data that provide a large span of well-distributed response values from a completed and relatively extensive preliminary screen.^11^

### Parameter analysis and processing

Proper parameter refinement can help simplify and improve the model interpretation.^105^ A preliminary necessary operation is parameter normalization, which is conventionally performed using eqn (1), where the mean is subtracted from the sample and then the resulting value is divided by the standard deviation.^106^ This procedure allows all parameters to possess the same scale and deviation, so that the coefficients in multivariate linear regression models are reflective of the variance accounted for by each parameter.1
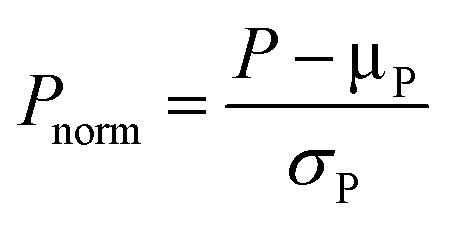



A parameter intercorrelation analysis through visualization of correlation matrices is highly desirable for several reasons. First of all, as the physical meaning of some parameters (*e.g.*, structural features) is unclear, it is beneficial to benchmark them against well-defined, experimentally-derived descriptors. Secondly, multicollinearity, where parameters have significant intercorrelations with each other, should preferably be avoided in multivariate correlations.^107^–^109^ When highly intercorrelated parameters coexist in the same model, the effective variance becomes associated with the difference between parameters. This causes the random noise in descriptor values to be amplified. Furthermore, the coefficient values can be erroneous, which damages the reliability of the model. As a result, it is vital to perform an intercorrelation analysis which helps avoid such collinear parameter selection. In a recent report by Guo *et al.*, a correlation map, an initial step in principal component analysis (PCA),^110^ was effectively utilized as a visualization tool to identify intercorrelations between parameters.^99^

If the study is entirely extrapolation-oriented, and the parameter set is considerable in size, a PCA is highly recommended.^110^–^113^ Such process analyzes the variation of the original parameter set, which then creates a new set of orthogonal parameters that can typically account for the vast majority of the variance with a considerably smaller number of parameters. This analysis is extensively applied to reduce dimensionality, which significantly improves the modelling efficiency as well as diminishes the concern for collinearity. However, it is not recommended if a mechanistically informative model is desired, as the reconstructed orthogonal parameters have less obvious meaning, and the resulting models can be difficult to interpret.

Notably, with the data being divided into training and validation sets, the standard for parameter processing (*e.g.*, means, standard deviations, and principal component directions) should all be established by the training set, with the validation set being processed accordingly, so that the external validation data does not directly impact the model composition.

### Subset design and univariate correlations

It is necessary to identify impactful features at an early stage of data analysis, which can be achieved through univariate correlation analysis on data subsets, where ideally, structures bearing significant similarities with each other provide a singular characteristic to be interrogated.^99^,^114^ The most relevant features identified through single-parameter analysis are not always directly applicable in the construction of multivariate models. However, apart from demonstrating the general trends, when combined with the intercorrelation analysis, univariate models can aid in interpreting the occasionally complicated comprehensive models.

### Preliminary multivariate model construction

This section is dedicated to the construction of a linear regression model on the basis of a free energy relationship analysis. Other statistical methods that are also effective for quantitative analysis yet less applied in the analysis of catalytic systems, such as random forest^115^,^116^ and artificial neural network,^117^–^119^ are not discussed in this review.

Least-squares linear regression by forward feature selection^120^,^121^ is a common method for model construction. Starting from either a constant term, or an initial guess of the model containing the presumed relevant parameters, this method evaluates the change in statistics caused by addition/removal of each parameter, and incorporates the most consequential term at each step, until no significant improvement can be found. Backward feature elimination has also been applied in several cases,^11^,^61^ where all parameters will be incorporated in the model at the beginning, and the algorithm reduces the variables by removing the insignificant terms.

The employment of weighted least squares, where the entries are not all treated equally but are instead weighted based on certain criteria, can also be desirable. For example, in extrapolative modelling of a system aimed at a highly enantioselective as well as high yielding process, where the accuracy is emphasized in the overall high-performance region, a yield/TOF-based weighting can be applied to the enantioselectivity model, so that the low-yielding reactions are considered less important. Another application of weighted least squares is that, in cases where the system is suspected to be plagued by a few outliers, the iteratively reweighted least squares (IRLS),^122^ where each entry is weighted based on its residual error, can be very useful in eliminating the influence of the outliers.

To avoid overfitting,^123^ where the model tries to explain all the random noise in the training set and makes it specific towards the training set with poor generalizability, the number of descriptors should be limited (empirically less than 1/3 of the number of entries).^124^ Furthermore, the following methods can be employed to validate the model.

### Model evaluation and optimization

Cross-validation and external validation are the most common methods for model verification. Both can be employed to test for the generalizability of the model. Cross-validation is performed internally on the training set, where part of the data is excluded and predicted based on a model with the same parameter combination reconstructed from the remaining set of data.^95^,^96^,^125^,^126^ The prediction accuracy can then indicate the stability and generalizability of the models. Leave-one-out cross-validation, where each point in the training set is removed and tested individually, is the only type which would provide a constant result, depicted as *Q*^2^, which is used as a common statistical measure.^127^ For other cross-validation options, it is common to average the results from multiple runs.

External validation, in contrast, deploys an additional set of data separated from the training set, whose empirical results are known before model development. The validation data set is often considered to be in between the training set, which is used for model construction, and test set, for which the prediction comes before the experimental results. It allows for a convenient evaluation of both the generalizability of the model, and the design of the training set.^97^ Ideally, provided an aptly orchestrated training set, it is adequate to adopt the rest of the existing data as external validations, despite the ratio of the two sets of data. Otherwise, with a rather random training/validation partition, the results could resemble a cross-validation within the entire dataset.

As a side note, multiple techniques have been developed to modify and improve the prediction accuracy of least squares regression models. For instance, LASSO regression, the restricted least squares method where coefficients for some parameters are reduced or set to zero, is used to decrease the prediction variance with slight sacrifice of model bias. Furthermore, the interpretability of models may also improve as a result of parameter elimination^128^

It is important to note that the standard for model evaluation would change based on the primary goal of the study. For purely extrapolative modelling, accuracy and generalizability are imperative, while complexity and obscurity of the models are not considered vital flaws. Conversely, for mechanistically informative modelling, high statistical measures sometimes have to give way to simplicity and interpretability, in which case reasonably reliable models composed of a small number of parameters with clear physical meaning can be more preferable over complicated models comprised with a large number of parameters including exponential and cross terms, albeit better performance of the latter.^129^,^130^ Additionally, for mechanistically-driven studies, the parameters should not be strongly interdependent, even with acceptable levels of noise amplification. The reason being that in such cases, the consequential features involved would be the differences between the parameters instead of the features described by any of them, leaving the models difficult to interpret.

### Model failures and solutions

It is not an uncommon scenario where no satisfactory model can be found. Listed here are some typical causes for model failure and possible solutions.

#### Change of reaction mechanism

**Fig. 13:**
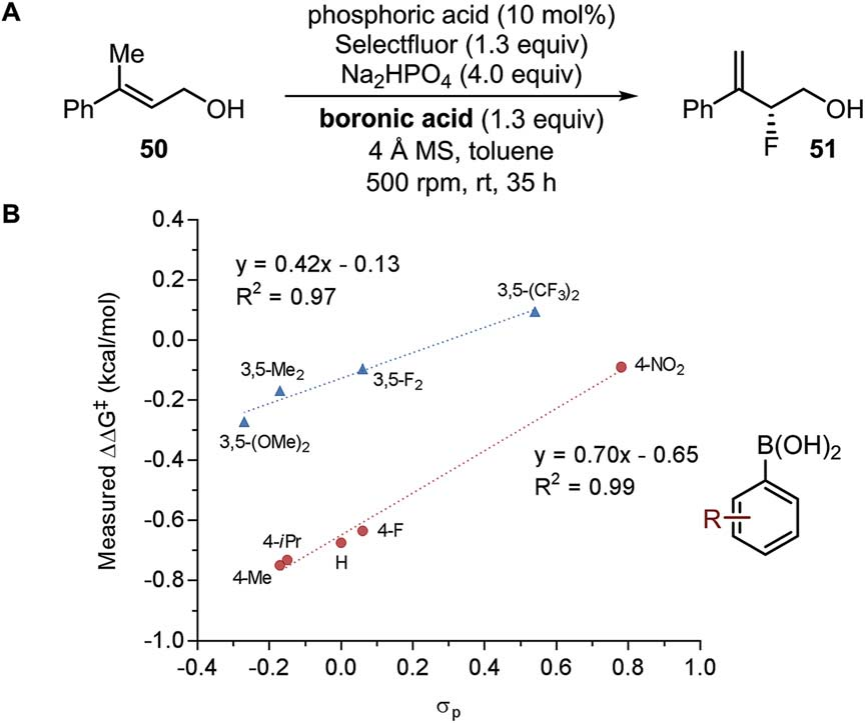
Fluorination of allylic alcohols.

It is difficult to build a comprehensive model for a system if there are multiple pathways leading to the products being analyzed. In this case, finding the features that describe the origin of mechanism change and dividing the data into subsets accordingly could provide access to a comprehensive model.^90^,^131^ As an example, Neel *et al.* reported an enantioselective fluorination reaction of allylic alcohol **50**, in which the Hammett correlation revealed an apparent change of mechanism as a function of the substitution pattern of boronic acid (Fig. 13).^131^ As a result, the system was divided accordingly, and modeled as two individual sets of data.^131^

#### Presence of outliers

If the majority of the dataset can be accurately described by an interpretable model, with a few exceptions (which can be recognized by performing a *t*-test on the residual errors), it would be reasonable to suspect an outlier scenario where the inability of the model in describing certain entries has chemistry-related causes. The common sources of outliers include occurrence of side reactions, decomposition of unstable structures, change of mechanism caused by distinct structural features,^132^ and problematic conformation of the parameter sources (*e.g.*, not the lowest in energy, or multiple low-energy conformations instead of one need to be accounted for). If the structures and/or features of the supposed outliers support the speculation, it would be proper to refine the parameters or remove the outliers.

#### Unrepresentative training set

Poorly designed training sets which are limited in diversity, range, being clustered, or containing outliers, can be ineffective in model construction. In this case, it is rational to redefine the training set.^98^ A scope extension is recommended if the diversity and/or range of the entire dataset is a concern.

#### Insufficient parameter space

If all former attempts fail, it is highly probable that the key molecular features affecting the process is not included in the parameter set, and new descriptors need to be explored to effectively describe the system under study. Tropsha and coworkers have developed a scoring system (MODelability Index, MODI) to evaluate the modelability of data sets.^133^ The system evaluates the extent to which similar structures afford comparable empirical outcomes, with ‘similarity’ determined through nearest neighbor analysis of descriptors. This algorithm reveals the ability of the current parameter set to address the effective diversity of the system under study.

## Model applications

To demonstrate the application of this modelling approach, two case studies will be discussed. In the first study, the MLR model was developed to identify a better performing catalyst while in the second example, the model was constructed to interrogate the mechanism and distinguish the underlying NCIs involved in the reaction.

### Virtual screening

Virtual screening is the classical application of reliable quantitative models.^30^ From an experimental standpoint, the practicality of synthesis and commercial availability of starting materials must be taken into consideration when designing the virtual screening deck. Notably, the structures to be evaluated should be within the generalizable region of the models where the molecular structures bear similarity with certain entries in the training set, as critical changes unaccounted for in the training set could lead to prediction failure. Remarkably, it has been observed that averaging the predictions from multiple reliable models can help improve the accuracy of estimations.^13^

#### Structure–enantioselectivity relationship of thiourea catalyst

**Fig. 14:**
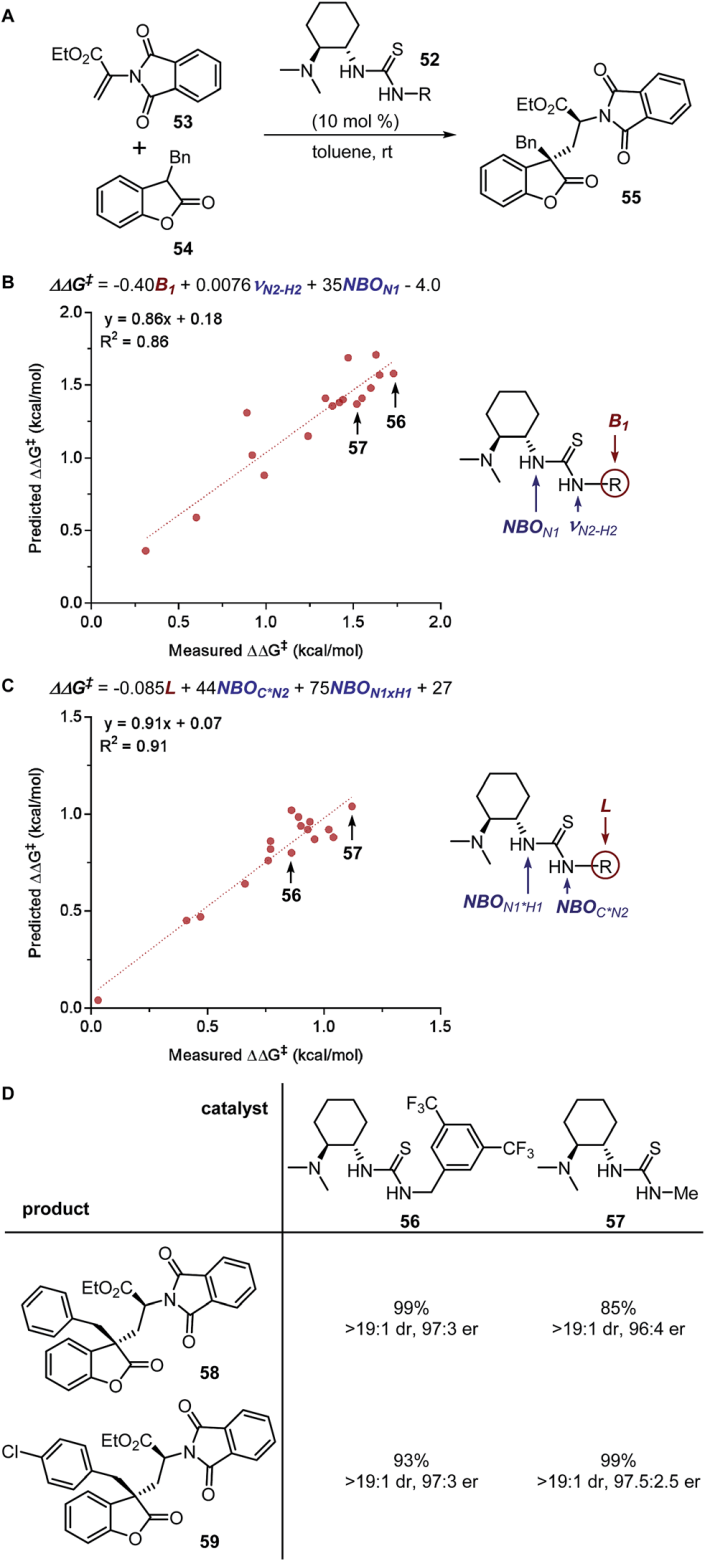
(A) Thiourea-catalysed asymmetric conjugate addition. (B) MLR model of enantioselectivity. (C) MLR model of diastereoselectivity. (D) Evaluation of optimal catalysts.

The multidimensional modelling approach was utilized by Li, Cheng, and coworkers to obtain predictive models that portray the thiourea catalyst **52** effects on the enantioselectivity as well as diastereoselectivity in the asymmetric conjugate addition reaction between 2-phthalimidoacrylate **53** and 3-substituted benzofuranone **54** (Fig. 14A).^134^ The resulting optimal models for enantioselectivity (Fig. 14B) and diastereoselectivity (Fig. 14C) indicated the need for small electron-withdrawing groups as catalyst substituents to achieve high enantioselectivity. Additionally, the utilization of the thiourea nitrogen NBO charge and IR N–H stretching frequency demonstrates the significance of the H-bond activation with the substrate. After further optimization of reaction conditions, two catalysts (3,5-trifluoromethylbenzyl **56** and methyl **57**), which were predicted according to the structure-selectivity model were evaluated experimentally with various 3-benzofuranones and alkyl 2-phthalimidoacrylates, both leading to high enantiomeric ratios (Fig. 14D). Further evaluation of the performance of bifunctional tertiary-amine hydrogen-bonding catalysts in Michael reactions demonstrated the requirement of less bulky *N*-substituents.^135^

### Predictor variable analysis

Mechanistic interpretation of the relevant parameters used as predictor variables in the models is a less common, yet highly advantageous application of the molecular-feature-based models. In addition to providing a mechanistic rationale for the observed chemical phenomenon, such analysis can efficiently guide virtual screening towards a more focused, smaller library of simulated structures. However, it is noteworthy that mechanistic interrogation based on predictor variable analysis can only be successfully performed if there is already a prior hypothesis for the reaction mechanism. Due to the unavoidable interrelationship between parameters, multiple statistically satisfactory models, where parameters can be substituted for each other, can be attained. Typically, models that consist of parameters with discernible physical meaning or correspond with existing mechanistic information are selected for further validation.

#### Mechanistic elucidation in enantiodivergent fluorination of allylic alcohols

**Fig. 15:**
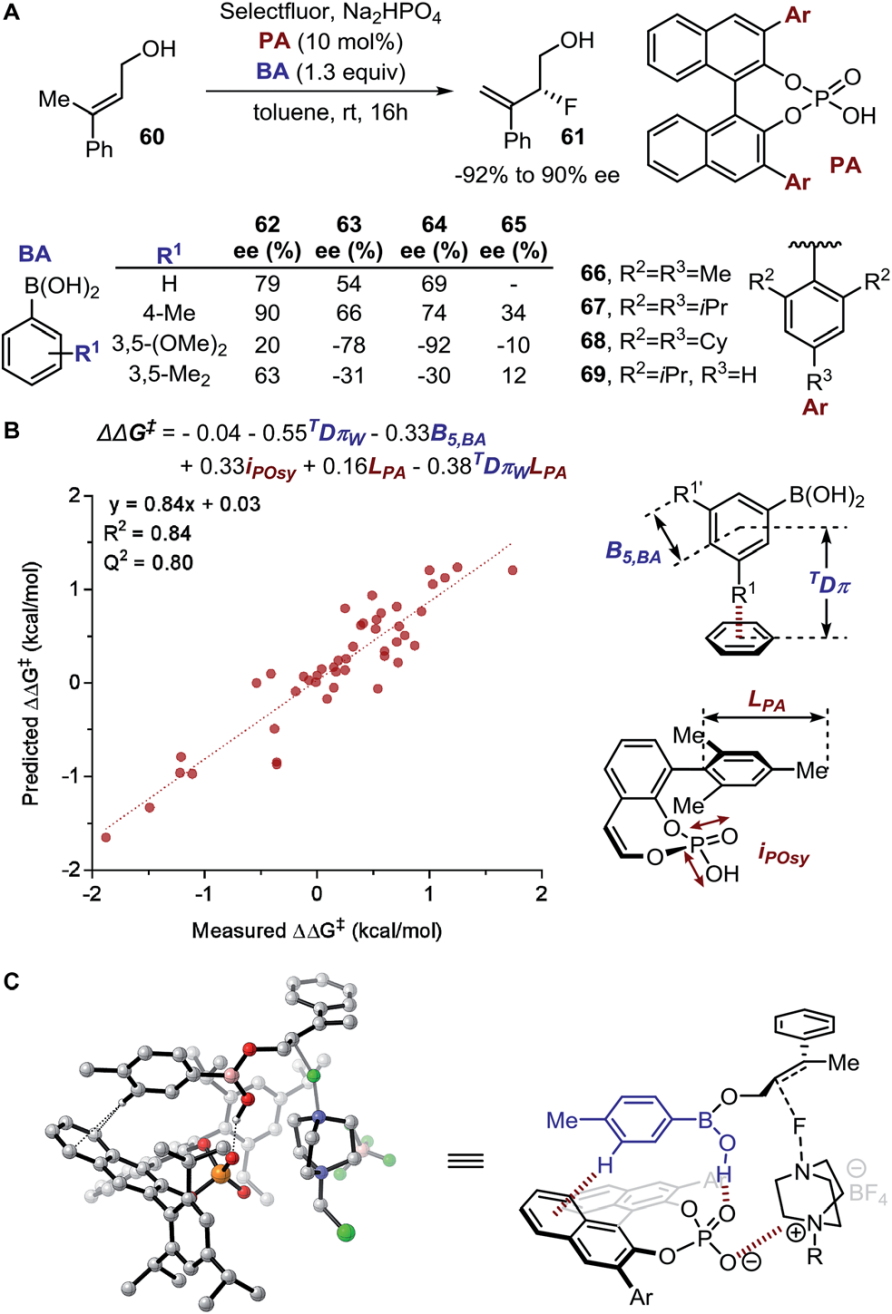
(A) Enantiodivergent fluorination of allylic alcohols. (B) Multivariate model of enantioselectivity. (C) Transition state analysis.

The enantiodivergent fluorination of allylic alcohol **60** exhibiting a ΔΔ*G*^‡^ range of 3.5 kcal mol^–1^ was demonstrated by Toste, Sigman, and coworkers to be a suitable reaction system for investigation of underlying NCIs relevant in controlling the observed enantioselectivity (Fig. 15A).^89^ Based on experimental results, it was proposed that a condensation reaction between the allylic alcohol and the boronic acid (BA) occurs to form a mixed boronic ester. In the enantiodetermining step, it was hypothesized based on the structures that an H-bond forms between the mixed boronic ester and the chiral phosphate anion (PA). Additionally, two key NCIs were hypothesized: (1) *meta*-substituted BAs resulted in inverted enantioselectivity and (2) PAs containing 2,6-disubstitutions resulted in greater sensitivity towards the BA substitutions. To probe the proposed NCI interactions, the *E*π_w_ and *D*π_w_ NCI parameters were calculated for each substituent. The NCI parameter *D*π_w_, describing the geometric readout to establish the T-shaped C–H π interaction, was found relevant in multivariate linear regression, along with the Sterimol parameters *B*_5,BA_ and *L*_PA_, defining the steric influence from the BA and the PA catalyst, respectively, and the symmetric stretching intensity *i*_Posy_, demonstrating the H-bonding and electrostatic interaction capability of each PA catalyst (Fig. 15B).

A computational transition state (TS) analysis was performed in order to clearly visualize the involved NCIs in the fluorination of allylic alcohols. As depicted in Fig. 15C, the T-shaped NCI indicated by the multivariate model was obtained from the DFT study of the transition state without intended pre-arrangement of structure. Additionally, analogous to the parameters obtained from the multivariate model, the BA *meta*-substituent and the PA binaphthyl moiety are involved in a T-shaped π interaction. Furthermore, the *D*π_w_ parameters obtained from the ground state calculations are consistent with the computed distances between the BA aryl ring and PA binaphthyl moiety observed in the TS.

## Conclusions

In summary, multivariate linear regression models utilizing physical organic molecular descriptors were demonstrated to be effective towards their application in virtual screening and mechanistic interrogation. Compelling reports that executed virtual screening led to acceleration of reaction optimization. Mechanistic interpretation of the structural meaning of these relevant parameters has contributed to the analysis of the observed chemical phenomenon. We hope that the presented detailed modern MLR model development protocol will serve as a guide for utilization of this approach.

## Conflicts of interest

The authors declare no conflict of interest.
